# Cholinergic-Induced Specific Oscillations in the Medial Prefrontal Cortex to Reverse Propofol Anesthesia

**DOI:** 10.3389/fnins.2021.664410

**Published:** 2021-05-26

**Authors:** Lieju Wang, Weijie Zhang, Ying Wu, Yibo Gao, Na Sun, Hao Ding, Jinxuan Ren, Lina Yu, Liangliang Wang, Fen Yang, Wang Xi, Min Yan

**Affiliations:** ^1^Department of Anesthesiology, The Second Affiliated Hospital, School of Medicine, Zhejiang University, Hangzhou, China; ^2^Department of Anesthesiology, Interdisciplinary Institute of Neuroscience and Technology, The Second Affiliated Hospital, School of Medicine, Zhejiang University, Hangzhou, China; ^3^Key Laboratory of Biomedical Engineering of Ministry of Education, College of Biomedical Engineering and Instrument Science, Zhejiang University, Hangzhou, China

**Keywords:** optogenetic, basal forebrain, cholinergic, glutamatergic, general anesthesia, emergence

## Abstract

General anesthesia is a drug-induced reversible state comprised of altered states of consciousness, amnesia, analgesia, and immobility. The medial frontal cortex (mPFC) has been discovered to modulate the level of consciousness through cholinergic and glutamatergic pathways. The optogenetic tools combined with *in vivo* electrophysiological recording were used to study the neural oscillatory modulation mechanisms in mPFC underlying the loss of consciousness (LOC) and emergence. We found that optogenetic activation of both cholinergic and glutamatergic neurons in the basal forebrain (BF) reversed the hypnotic effect of propofol and accelerated the emergence from propofol-induced unconsciousness. The cholinergic light-activation during propofol anesthesia increased the power in the β (12–20 Hz) and low γ (20–30 Hz) bands. Conversely, glutamatergic activation increased the power at less specific broad (1–150 Hz) bands. The cholinergic-induced alteration to specific power bands after LOC had opposite effects to that of propofol. These results suggested that the cholinergic system might act on more specific cortical neural circuits related to propofol anesthesia.

## Introduction

General anesthesia is a reversible, anesthetic drug-induced state in which patients undergo an amalgamation of altered states of consciousness, analgesia, amnesia, and immobility (Brown et al., 2011). One of the biggest mysteries of modern medicine is how anesthetic drugs induce unconsciousness and how patients subsequently recover from general anesthesia (Kennedy and Norman, 2005). Neural oscillatory dynamics that are readily visible in the physiological measurements (electroencephalogram, EEG, and local field potential, LFP) are used to empirically characterize the anesthesia state (Ching and Brown, 2014). Different anesthetics induced altered specific oscillation band changes during the loss of consciousness (LOC) because of the discrepant molecular targets in the brain (Blain-Moraes et al., 2014, 2015). Propofol, one of the GABA_*A*_ receptor agonist anesthetics, exhibited specific activation of the delta band and theta-gamma coupling during the LOC and emergence, and is commonly used in clinical trials (Breshears et al., 2010). However, due to the lack of current literature on the formation of propofol-induced oscillation change in microcircuits, a detailed understanding of the neural mechanism is warranted.

Recent studies suggest that both LOC and emergence from general anesthesia is brought about by the modulation of ascending arousal systems, such as glutamatergic and cholinergic systems, in the central nervous system (Ren et al., 2018; Wang et al., 2019). It is widely accepted that acetylcholine (Ach) in the cortex is predominately derived from cells located in the basal forebrain (BF) (Zant et al., 2016). Moreover, BF cholinergic neurons have been shown to play an imperative role in the sleep-wake cycle transition (Han et al., 2014). Selective modulation of the BF cholinergic neurons has been found to change the sedative potency of general anesthetics and the duration of loss of the righting reflex (LORR) during anesthesia (Laalou et al., 2008; Leung et al., 2011; Luo et al., 2020). Intriguingly, systematic administration of physostigmine promoted arousal in human patients during propofol anesthesia (Xie et al., 2011). BF cholinergic neurons strongly innervate the medial prefrontal cortex (mPFC) to partly exert the wake-promoting effect (Bloem et al., 2014; Ahrlund-Richter et al., 2019). The BF cholinergic activation in the prefrontal cortex has been also demonstrated to contribute with paramount value in the depth of consciousness (Pal et al., 2018). However, the characteristics and functions of the pathways projecting from the BF to mPFC on the altered states of consciousness induced by propofol remain to be elaborated.

The BF innervating the frontal cortex contains glutamatergic neurons (∼55%), as well as cholinergic neurons (∼10%) (Gritti et al., 2006). Previous studies have shown that glutamatergic neurons in the BF regulate the sleep-wake cycle (Xu et al., 2015; Peng et al., 2020). However, there is no evidence showing the role of glutamatergic neurons in the BF on the altered states of consciousness induced by general anesthesia.

To investigate these questions, we used optogenetic activation of two different neurotransmitters, cholinergic and glutamatergic in the BF, to underlie the mPFC oscillatory mechanisms of altered states of consciousness induced by propofol general anesthesia.

## Materials and Methods

### Animals

Adult wild-type (6–8 weeks old) C57BL/6 mice and ChAT-ChR2-EYFP transgenic mice (ChAT-ChR2-EYFP mice as a generous gift from Prof. Duan Shuming, Institute of Neuroscience, School of Medicine, Zhejiang University) were used. During the experiment procedures, all animals were given water and regular mice chow *ad libitum* and housed individually under climate-controlled conditions with a 12-h light/dark cycle, with lights on at 7:00 AM. The temperature in the room was maintained at 21–23°C. All the procedures were conducted according to guidelines approved by the Animal Care Committee of the Zhejiang University (Hang Zhou, Zhejiang, China).

### Virus Injection

Wild-type C57BL/6 mice were anesthetized with sodium pentobarbital (1% wt/vol) and AAV-CaMKIIα-hChR2 (H134R)-mCherry virus (Shumi Technology, Wuhan, China) was bilaterally injected into the BF (AP = −0.6; ML = 0.8; DV = −4.8). We injected 0.1–0.3 μl of the virus into each location at 0.01–0.03 μl/min. The syringe was not removed until 15–20 min after the end of infusion to allow the diffusion of the virus. After injection, mice were allowed 2–3 weeks for recovery and virus expression.

### LFP Recording

Mice were deeply anesthetized with sodium pentobarbital (induction 1% wt/vol) and fixed in a stereotaxic device (RWD, China). After exposing, cleaning, and disinfecting the skull bone, four electrodes made by nickel chromium(California Fine Wire, United States)were implanted. Targeting the mPFC (AP = 1.5; ML = 1; DV = −1.5, Atlas of Paxinos and Watson), two screw electrodes were fixed into the frontal (AP = 2; ML = 1) and parietal (AP = −3; ML = 1.5) cranium for grounding. Mice were allowed to recover for at least 7 days. Continuous LFP was recorded at 1 KHz using Central Acquisition system (Cerebus system, Blackrock Technology, United States). The LFP signals were amplified and filtered (0.5–500 Hz) for further analysis. The LFP recording was sustained for 15 min before and after propofol delivery.

### Light Stimulation

Optical fibers were implanted into the BF (AP = −0.6; ML = 0.8; DV = −4.8) according to the Atlas of Paxinos and Watson of previous work in the ChAT-ChR2-EYFP mice (Han et al., 2014). For light stimulation, the optical fibers were bilaterally implanted in BF. Laser light was generated using a fiber-coupled 473 nm solid-state laser diode (473 nm, Lasercentury, Shanghai, China) and was delivered *via* the ceramic ferrule. Laser light stimulation was driven by software-generated TTL pulses (10 ms@20 Hz for 5/30 s for 30 min post propofol) (Anilab, Ningbo, China) (Han et al., 2014; Peng et al., 2020). Wild-type mice with optical fibers implanted served as control mice. For electrophysiology data analysis, the TTL pulse was also recorded by the Cerebus recording system simultaneously.

### Anesthetic Performance During Propofol

LORR was used as the behavioral time-point to investigate the hypnotic properties of propofol (AstraZeneca, United Kingdom), following previously described methods with slight modifications (Leung et al., 2013). To determine the propofol dosage required to induce LORR, an initial bolus of 50 mg/kg was given to the mouse intraperitoneally. 15 min absorption time was set after injection. LORR was considered if there were two failed attempts to right itself (four paws grounded) within 30 s after being placed supinely. Subsequently, recurring 25 mg/kg was administered and retested until LORR was achieved. The percentage of mice showing LORR at each dose of propofol was established in the control and optical groups, and the ED_50_ (50% effective dose) and ED95 (95% effective dose) values of propofol were estimated from the dose-response equation described in **Statistical Analysis**. The time to LORR and time for recovery of righting were investigated with 200 mg/kg propofol (ED_95_), in order to assess propofol induction and emergence time.

### Histology

To verify the validity of the fibers’ placement and virus expression, the mice were perfused with saline and a 4% paraformaldehyde in 0.1 M PBS. After perfusing, the brains were removed, post-fixed overnight in 4% paraformaldehyde, and then immersed in a 30% sucrose solution at 4°C for cryoprotection. 30 μm-thick coronal slices were collected and stored in PBS at 4°C. Finally, the sections were rinsed in 90% glycerol, cover slipped, and imaged by the fluorescence microscope (Olympus VS 120, Japan).

### Statistical Analysis

All values are shown as mean ± SEM. Statistical analyses were performed using Graphpad Prism (version 5.01, Graphpad Prism, Inc., San Diego, CA, United States). LORR dose-response data were curve-fitted by non-linear regression with Prism to give the half-maximal effective concentration–dose values (ED_50_ ± SEM) with the equation Y = Y_min_ + (Y_max_ − Y_*min*_)/[1 + 10 ^*log(ED50* – *X)* × m^], where Y is the percentage of the population showing LORR, Y_min_ and Y_max_ are the minimal and maximal values of Y, respectively, ED_50_ is the drug dose for a half (Y_max_ − Y_min_), X is the logarithmic drug dose, and m is the Hill slope constant. The F-test for non-linear regressions was then used to determine whether the calculated ED_50_ was significantly different between groups. At the time of the onset of LORR, the recovery of righting reflex (RORR) was compared using an unpaired Student *t*-test.

For LFP data analyses, data were obtained from the mPFC at 200 mg/kg propofol. The recorded signals were pre-filtered with Matlab 2010 (Mathworks, United States) to exclude artifacts. All the 15 min data were used to plot spectrogram using FFT multi-taper function of the MATLAB chronux toolbox^1^. For spectrogram analysis, the spectrogram data at the initial 100 s when the mice were awake were used as the baseline reference to calculate the mean and standard deviation for normalizing [Normalized *Z* = (the point value -mean)/Standard deviation]. After spectrogram normalization, the laser-stimuli-triggered spectrogram changes were averaged from all the stimuli in 15 min recording period (*n* = 36). For comparisons of spectrogram power 5 s before and after light stimulation, we used one-way repeated-measures ANOVA, followed by Bonferroni *post hoc* tests. *P* value less than 0.05 (two-tailed) is considered to be statistically significant.

## Results

### The Activation of BF^*Ach*^→mPFC on Propofol Anesthesia

**FIGURE 1:**
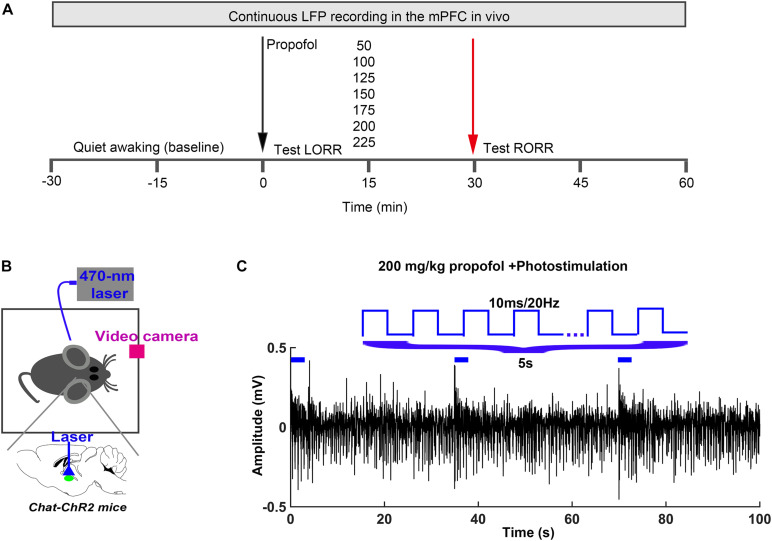
Experimental procedures **(A)** *In vivo* LFP recording and behavioral test timeline. **(B)** Schematic of optogenetic stimulation of ChR2-expressing BF cholinergic neurons. **(C)** The parameter of light stimulation during the *in vivo* recording.

**FIGURE 2:**
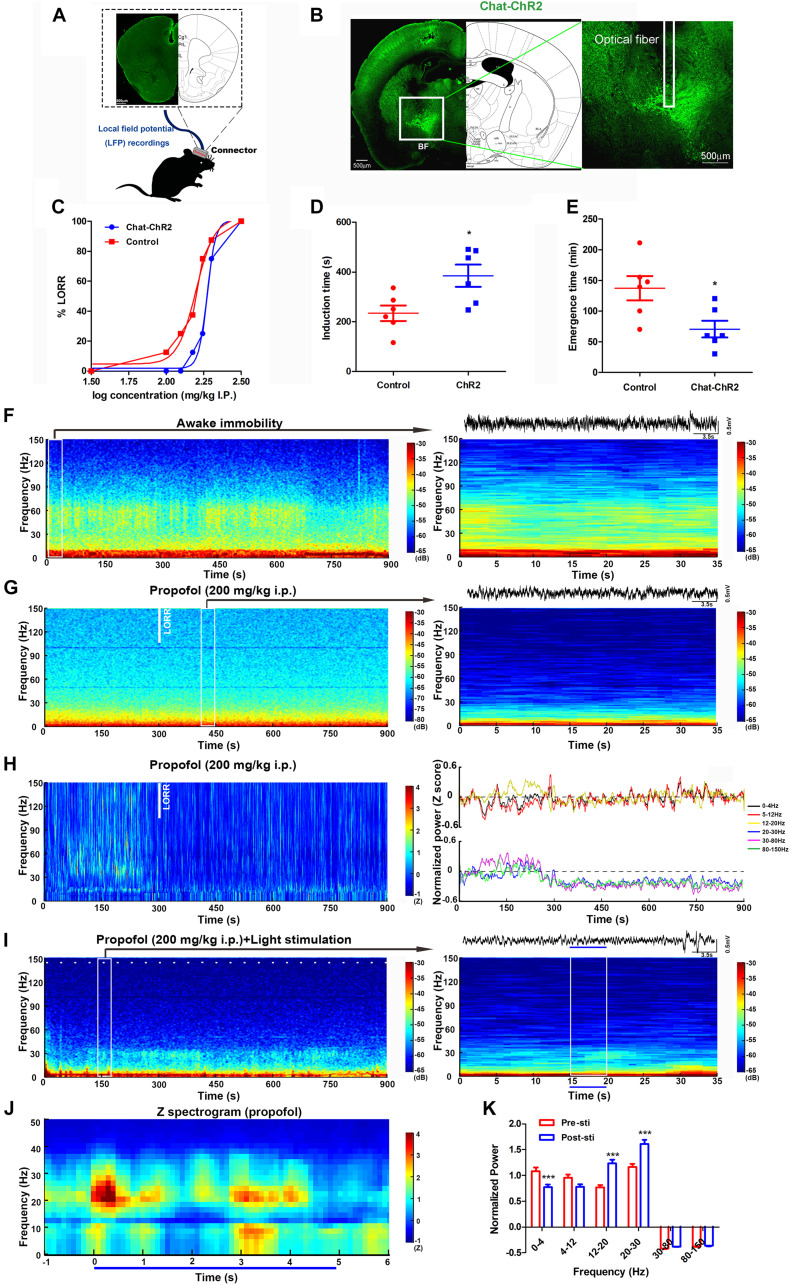
Optogenetic activation of cholinergic neurons in the BF reversed propofol anesthesia with mPFC LFP change. **(A)** The location of electrodes embedded in the mPFC. **(B)** Typical placement of optical fibers over the injection site and ChR2-expressing cholinergic neurons. **(C)** The dose-response curve plotted from the proportion of mice losing the righting reflex under graded propofol (*n* = 12 in both groups). Induction **(D)** and emergence **(E)** time after stimulation of BF cholinergic neurons when exposed to propofol at the dosage of 200 mg/kg (*n* = 6 both). ^∗^*P* < 0.05, compared with the control group. **(F)** Typical mouse spectrogram during the awaking state (left), and an enlarged, 35 s spectrogram with raw LFP data (right). **(G)** 15-min spectrogram (left) and a 35 s enlarged time window with raw LFP data (right) from a typical mouse during propofol anesthesia. **(H)** Normalized spectrogram averaged from six mice (left) and power differential at 0–4, 5–12, 12–20, 20–30, 30–80, and 80–150 Hz (right). **(I)** 15-min spectrogram (left) and an enlarged 35-s time window with raw LFP data from a typical mouse during propofol anesthesia with light stimulation (right). White dashes (left) and blue line (right) represent light stimulation. **(J)** Normalized spectrogram computed from nine mice under light stimulation during propofol at 200 mg/kg, trial = 52). **(K)** *Post hoc* analysis of the power at 0–4, 5–12, 12–20, 20–30, 30–80, and 80–150 Hz for 5 s before and after light stimulation during propofol at 200 mg/kg. “0” means the onset of propofol administration in the Figures **(G,H,I)**. ^∗∗∗^*P* < 0.001, compared with the pre-sti group at every frequency range.

To investigate the cholinergic function during the propofol-induced anesthesia, we firstly performed an optogenetic activation of the BF cholinergic neurons during propofol-induced anesthesia in mice with graded propofol (Figure 1A). The behavior response of the proportion of mice LORR, induction time, and emergence time (LORR to RORR), were recorded by synchronous video recording (Figure 1B). After intraperitoneal injection (i.p.) of propofol, a 473 nm blue light (10 ms@20 Hz, for 5/30 s) was continuously delivered to the BF of a ChAT-ChR2-EGFP(ChAT) transgenic mouse for 15 min (Figure 1C). The typical placement of the electrodes, fibers, and the expression of ChAT were shown in the Figures 2A,B. The sensitivity of ChAT mice to propofol was significantly reduced by the stimulation of light, characterized by a rightward shift to the dose-response curve (Figure 2C). The effective dose instigating 50% (i.e., ED_50_) of the LORR in the wild-type mice was 157.7 mg/kg (95% CI, 138.3–179.8 mg/kg, and *n* = 12), and was significantly different (*P* = 0.0008) to ChAT mice with 197.4 mg/kg (95% CI, 178.4–206.9 mg/kg, and *n* = 12) (Figure 2C). Based on the ED_95_ value, we chose the 200 mg/kg propofol dose to test LORR and the emergence time. The LORR was significantly prolonged following the light activation of BF cholinergic neurons in ChAT mice, compared with wild-type mice (*P* < 0.05, *n* = 6 for WT, and ChAT, respectively) (Figure 2D). The emergence time was significantly reduced by light stimulation (*P* < 0.05, *n* = 6 for WT, and ChAT, respectively) (Figure 2E). These results showed that selective activation of cholinergic neurons in the BF not only delays the time to unconsciousness but also promotes emergence from propofol anesthesia.

Brain network oscillations are ubiquitous in mammals. They are assumed to be an imperative signature for various cognitive abilities, such as learning (Brickwedde et al., 2019), memory (Liu et al., 2013), attention (ElShafei et al., 2019), and consciousness (Mukamel et al., 2014). Understanding the mechanisms and functions of these oscillations is necessary to understand how the brain carries out complex functions. During general anesthesia, the most practical technique for tracking the various states of the brain is the EEG/LFP, which measures scalp/local electrical potentials generated by cortical oscillations. The effects of propofol anesthesia on macroscopic dynamics are noticeable in EEG readings, which display several stereotypical oscillation patterns, including increased delta (0.5–4 Hz) power, decreased gamma (25–40 Hz) power, and an alpha (∼10 Hz) rhythm that is coherent across the frontal cortex (Lewis et al., 2012). Here, we placed the electrodes into the mPFC to acquire the LFP oscillations during the awake-immobility state and 200 mg/kg propofol administration (Figures 2F,G). During the immobility state, low-voltage, high-frequency activity was recorded in the mPFC, with high power over 30 Hz (Figure 2F). After exposure to the propofol at a dosage of 200 mg/kg i.p., an apparent increase in slow activity in the mPFC was observed, with comparatively lower power over 30 Hz (Figure 2G). After an average of 300 s LORR time, there was an increase in the delta (0–4 Hz) and theta (5–12 Hz) range and a decrease in power at higher frequencies (12–20, 20–30, 30–80, and 80–150 Hz) (*n* = 7, Figure 2H, left).

To understand the cortical neural oscillation dynamic changes during the BF cholinergic stimuli, we also recorded the LFP of the mPFC during light stimulation in the same region. The power changes occurred in frequencies between 12 and 30 Hz in one typical mouse during the light stimuli within propofol administration (Figure 2I). The averaged normalized spectrograms during 5 s light stimulation under propofol-induced anesthesia showed a 20–30 Hz power increase (Figure 2J). The light stimulation effectively decreased power in the delta (0–4 Hz) band and increased the power in the beta (12–20 Hz) and low gamma (20–30 Hz) bands (*P* < 0.001, *P* < 0.001, and *P* < 0.001, respectively) (Figure 2K). These alterations to specific bands were inversely identical to propofol-induced power changes after LOC in Figure 2H. The neural mechanism of propofol-induced LOC is associated to the BF-mPFC cholinergic ascending projection system.

### The Activation of BF^*Glu*^→mPFC on Propofol Anesthesia

**FIGURE 3:**
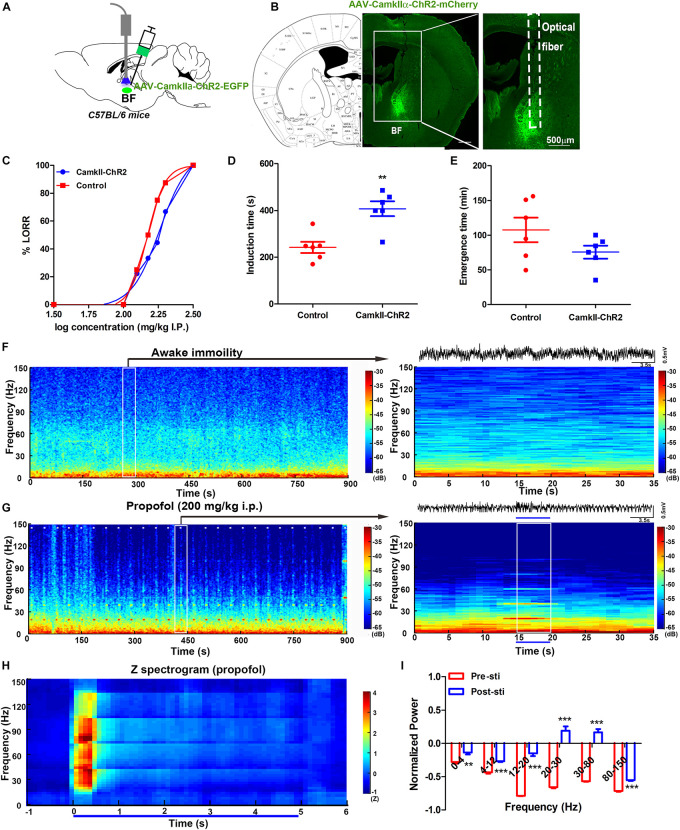
Selective activation of glutamatergic neurons in the BF reversed propofol anesthesia with mPFC LFP change. **(A)** Schematic of optogenetic activation of BF glutamatergic neurons. **(B)** Selective expression of the AAV-CamKII-ChR2 virus in the BF glutamatergic neurons. **(C)** Dose-response curve from graded propofol after light stimulation (10 mice in both groups). Induction **(D)** and emergence **(E)** time after stimulation of BF glutamatergic neurons when exposed to propofol at a dose of 200 mg/kg (six mice in both). ^∗∗^*P* < 0.01, compared with the control group. **(F)** Typical mouse spectrogram during the quiet awaking state for 15 min (left) and an enlarged 35 s time window with raw LFP data(right). **(G)** Typical mouse spectrogram during propofol anesthesia, accompanied by light stimulation for 15 min (left) and 35 s time window with raw LFP data (right), white dashes (left) and the blue line (right) represent light stimulation. **(H)** Normalized spectrogram computed from six mice under light stimulation during propofol at 200 mg/kg trial = 51). **(I)** *Post hoc* analyses at 0–4, 5–12, 12–20, 20–30, 30–80, and 80–150 Hz for 5 s before and after light stimulation during propofol at 200 mg/kg. “0” means the onset of propofol administration in the Figures **(F,G)**. ^∗∗^*P* < 0.001 and ^∗∗∗^*P* < 0.001, compared with the pre-sti group at every frequency range.

To investigate the glutamate function in BF, we bilaterally injected the virus AAV-CamKIIα-ChR2-mCherry into the BF of the C57BL/6 mice (CamKIIα) 2–3 weeks before behavioral testing (Figure 3A). The optical fibers were bilaterally implanted over the injection sites (Figure 3B). A blue light was delivered with the same parameters as previously used at a physiologically relevant frequency of 20 Hz (Supplementary Figure 1) (Xu et al., 2015; Peng et al., 2020). Compared with the control group, light stimulation of CamKIIα mice reduced their sensitivity to propofol anesthesia, characterized by a significant right shift of the dose-response curve (Figure 3C). ED_50_ in the control group was 148.3 mg/kg (95% CI, 138.9–158.3 mg/kg, and *n* = 10). In contrast, in the CamKIIα group, the ED_50_ (182.8 mg/kg, 95% CI, 147.2–227.2 mg/kg, and *n* = 10) was significantly increased (Figure 3C). The ED_95_ of 200 mg/kg propofol i.p. was selected to test LORR and the emergence time. The induction time showed a significant increase between the CamKIIα and wild-type groups (*P* < 0.05, *n* = 6 for WT and CamKIIα groups, respectively) (Figure 3D). The emergence time of the CamKIIα group in response showed a decrease, but this was not significant compared with the control group (*P* > 0.05, *n* = 6 for WT and CamKIIα, respectively) (Figure 3E). Taken together, these results indicate that modulation of glutamatergic neurons in the BF can also reverse propofol-induced unconsciousness.

We also recorded the LFP in the mPFC during light stimulation of BF glutamatergic neurons. The oscillation characteristics in the awake immobility were found to be identical to that in the ChAT mice (Figure 3F). Obvious power changes could be seen during all the light stimulation across 15 min recording under propofol anesthesia (Figure 3G, left). Selective light activation of glutamatergic neurons in the BF induced a shift from a low-frequency, high-amplitude slow oscillatory pattern to an active high-frequency, low-amplitude pattern (Figure 3G, right). The normalized averaged power showed a transient increase in the 20–80 Hz bands during the light stimulation (Figure 3H). A further *post hoc* Bonferroni test indicated that the 5 s light stimulation induced a significant increase in all bands during propofol anesthesia compared with that before the light stimulation (Figure 3I). Compared with the previous cholinergic activation-induced oscillation bands change, the glutamate activation showed more broad band changes during the propofol-induced general anesthesia, which suggested that the underlying neural mechanism may be different.

## Discussion

In summary, we demonstrated that the selective activation of both cholinergic and glutamatergic neurons in the BF could reverse the hypnotic effect of propofol. Additionally, they promote emergence with activation of alternative mPFC oscillation bands. Propofol induced specific power increases at 12–20 Hz during the wake-LOC state transition. This pattern appears similar to that observed in the human scalp EEG, characterized by broad-band β oscillations that coalesce into α oscillations after losing consciousness (Vertes, 2002; Flores et al., 2017). Selective activation of BF cholinergic neurons significantly decreased the delta power, which is an index to the unconsciousness state (Flores et al., 2017), but increased the power at 12–20 and 20–30 Hz, which was thought to antagonize the anesthesia-promoting effect of propofol in the mPFC. However, promoting the glutamate system could induce a systematic non-targeted change throughout all frequencies, suggesting the complex brain networks involving in the activation of the pathway projection may not have specificity as a cholinergic system. Moreover, previous work demonstrated that distinct consciousness patterns could be induced by different neuropharmacological agents (Kenny et al., 2016). The level of consciousness could be dissociated from cholinergic, behavioral levels, and neurophysiologic oscillations (Pal et al., 2020). These findings might explain the difference in cortical activation between cholinergic and glutamatergic neurons induced in our study. Indeed, the glutamatergic and cholinergic neurons in the BF project across the cortex, including the mPFC, but there is no comparison with any other cortical node (e.g., posterior parietal cortex) to understand if there is anything unique about the oscillations, which is a limitation of our study. Nevertheless, our findings suggest that cholinergic and glutamatergic arousal projections from the BF are sufficient to induce emergence in the mPFC from general anesthesia. Activating the cholinergic systems may modulate specific conscious related circuits, can provide a novel approach to accelerating recovery from general anesthesia, and treat or eliminate consciousness-related disorders such as hypoxia, postoperative delirium, and cognitive dysfunction.

## Data Availability Statement

The raw data supporting the conclusions of this article will be made available by the authors, without undue reservation.

## Ethics Statement

The animal study was reviewed and approved by Animal Care Committee of the Zhejiang University.

## Author Contributions

LeW, MY, and WX conceived the project and wrote the manuscript with input from all co-authors. WZ, YW, YG, and NS provided computational support. HD, JR, LY, LaW, and FY supported mouse work. All authors contributed to the article and approved the submitted version.

## Conflict of Interest

The authors declare that the research was conducted in the absence of any commercial or financial relationships that could be construed as a potential conflict of interest.

## Footnotes

1 http://chronux.org/
