# Supplementary material for: Cholinergic-Induced Specific Oscillations in the Medial Prefrontal Cortex to Reverse Propofol Anesthesia
Source: Front Neurosci. 2021 May 26;15:664410. doi: 10.3389/fnins.2021.664410 (PMC8187623; doi:10.3389/fnins.2021.664410)
Supplement: Supplementary file 1 [file Data_Sheet_1.docx]

***In vitro* electrophysiological recordings**

The slides were transported in room temperature to a recording chamber on the stage of a fluorescence microscope (BX51WI, Olympus) and preserved and flushed with aCSF. A micropipette puller was used for making Patch electrodes (P-97, Sutter Instruments) from borosilicate tubes (GC150-10, Harvard Apparatus). The pipettes (5-12 MΩ) contained (in mM): 97.5 K-gluconate, 1 MgCl2.6H2O, 32.5 KCl, 40 HEPES, 2 Mg-ATP, 0.5 Na-GTP and 0.5 EGTA, pH 7.4. MutiClamp 700B amplifier made Recordings (Molecular Devices). Neurons were identified as glutamatergic with expression of EYFP fluorescence and electrophysiological recording were made. Neurons were kept at -70 mV for voltage-clamp recordings. Signals were low-pass filtered at 10 kHz and digitized at 10 kHz (MICRO3 1401, Cambridge Electronic Design). Spike2 7.04 software were used for acquisition and analyzes of data (Cambridge Electronic Design). An optical fiber (200 µm diameter) was coupled to a 473-nm solid-state laser diode for neuronal light stimulation, (IKE-473-200T, Ikecool Corp.). The fiber was threaded through a stainless-steel sleeve (inner diameter 250 µm, outer diameter 480 µm) and fixated with adhesive (AC-001, Aron alpha). The fiber tip was clipped and refined, placed in aCSF, and inserted on top of the stimulation site. A pulse stimulator controlled the blue light (Master-8, A.M.P.I.). A power meter (PM10, Coherent) was used for measurement of the laser (0.5-1.5 mW) before experiments.

**Immunohistochemistry**

Adult mice were deeply anesthetized with Nembutal (400 mg/kg, i.p.) and transcardially perfused with chilled normal saline trailed by 4% paraformaldehyde in 0.1 M PBS. The brain was post-fixed for 2 hours and then cryoprotected in 30% sucrose. After surrounded in OCT compound, the brain was sectioned coronally at 50 μm on a freezing microtome (CM 1950, Leica). Sections were rinsed with 0.5% Triton-X in 0.1 M PBS and blocked with 3% normal bovine serum for 1 hour. Sections were incubated with primary antibody for glutamate immunohistochemistry (rabbit anti-Glu, 1:1000, Sigma G6642) in 0.1 M PBS for overnight at 4°C. Sections were then rinsed and incubated with Cy3-conjugated donkey anti-rabbit antibody (1:500, Jackson ImmunoResearch; 3 hours at room temperature). The analysis of immunostained neurons was performed immediately to avoid fading of fluorescence.

**Supplemental Figure legend**

**Supplementary figure 1** Characteristics of CAMKIIα-ChR2-EYFP expressing neurons *in vitro*.

(A) Diagram of BF location with light stimuli. (B) ChR2-expressing neurons (Green) in the BF with post hoc fluorescence in situ hybridization for the glutamate antibody (Red). bars: top left, 200 μm, others, 50 μm. Whole-cell recordings displaying BF glutamatergic neurons response to optical stimulation with the parameter of 5Hz/2ms (C) and 20Hz/10ms (D).
