# Supplementary figures and images for: Cholinergic-Induced Specific Oscillations in the Medial Prefrontal Cortex to Reverse Propofol Anesthesia
Source: Front Neurosci. 2021 May 26;15:664410. doi: 10.3389/fnins.2021.664410 (PMC8187623; doi:10.3389/fnins.2021.664410)

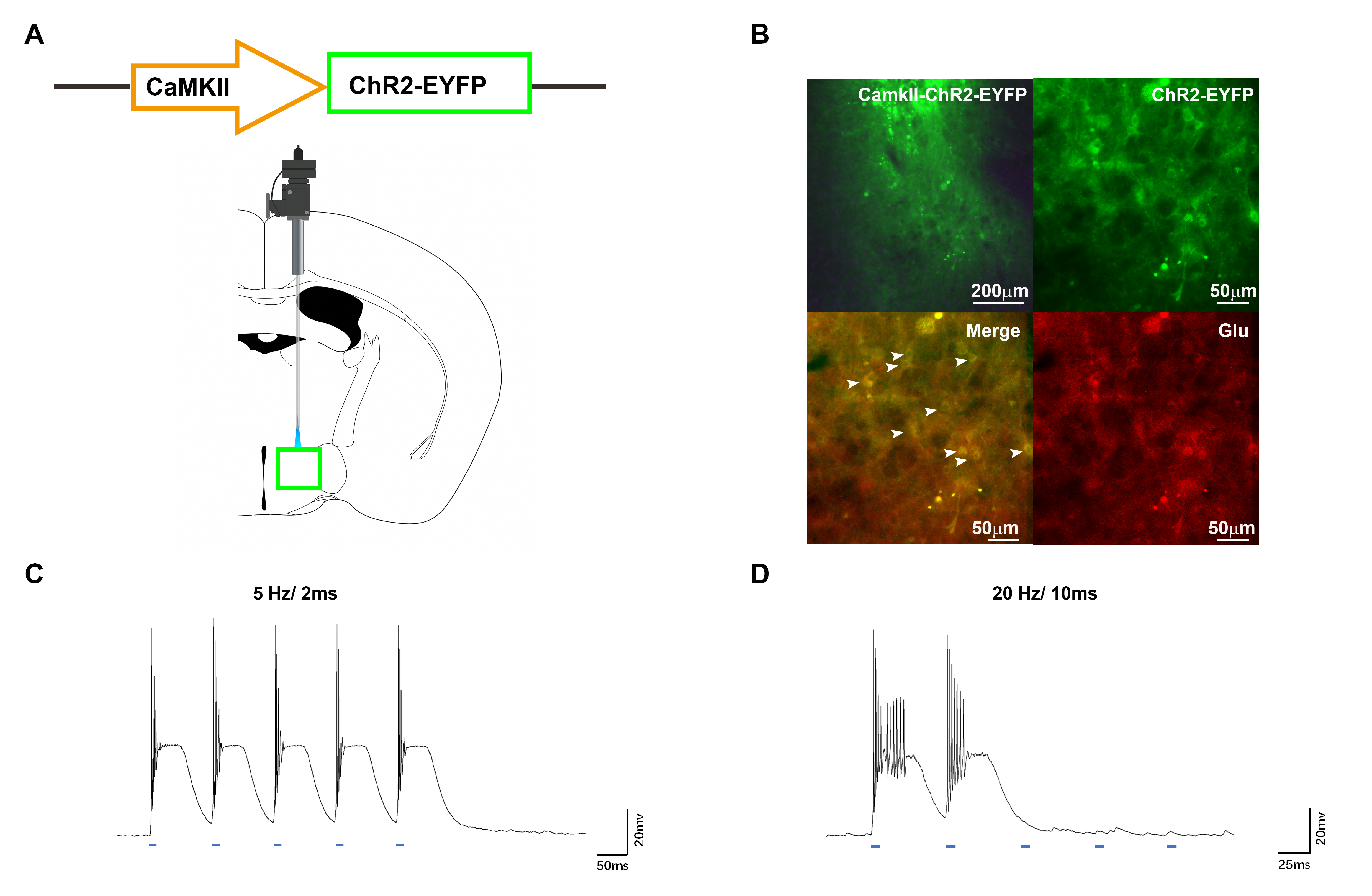

Supplement: Supplementary file 2 [file Image_1.TIF]
